# Pro-Inflammatory Characteristics of Extracellular Vesicles in the Vitreous of Type 2 Diabetic Patients

**DOI:** 10.3390/biomedicines12092053

**Published:** 2024-09-10

**Authors:** Shengshuai Shan, Abdulaziz H. Alanazi, Yohan Han, Duo Zhang, Yutao Liu, S. Priya Narayanan, Payaningal R. Somanath

**Affiliations:** 1 Clinical and Experimental Therapeutics, University of Georgia, Augusta, GA 30912, USA; 2 James and Jean Culver Vision Discovery Institute, Augusta University, Augusta, GA 30912, USA; 3 Department of Clinical Practice, College of Pharmacy, Northern Border University, Rafha 76313, Saudi Arabia; 4 Department of Microbiology, Wonkwang University School of Medicine, Iksan 54538, Republic of Korea; 5 Department of Cellular Biology and Anatomy, Augusta University, Augusta, GA 30912, USA

**Keywords:** diabetes, retinopathy, extracellular vesicles, claudin-5, inflammation

## Abstract

Diabetic retinopathy (DR) is a leading cause of blindness, yet its molecular mechanisms are unclear. Extracellular vesicles (EVs) contribute to dysfunction in DR, but the characteristics and functions of vitreous EVs are unclear. This study investigated the inflammatory properties of type 2 diabetic (db) vitreous EVs. EVs isolated from the vitreous of db and non-db donors were used for nanoparticle tracking analysis (NTA), transmission electron microscopy (TEM), immunogold staining, Western blotting, and proteomic analysis by mass spectrometry. Intracellular uptake of vitreous EVs by differentiated macrophages was evaluated using ExoGlow membrane labeling, and the impact of EVs on macrophage (THP-1) activation was assessed by cytokine levels using RT-qPCR. NTA and TEM analysis of db and non-db vitreous EVs showed non-aggregated EVs with a heterogeneous size range below 200 nm. Western blot detected EV markers (Alix, Annexin V, HSP70, and Flotillin 1) and an upregulation of Cldn5 in db EVs. While the db EVs were incorporated into macrophages, treatment of THP-1 cells with db EVs significantly increased mRNA levels of TNFα and IL-1β compared to non-db EVs. Proteomic and gene enrichment analysis indicated pro-inflammatory characteristics of db EVs. Our results suggest a potential involvement of EC-derived Cldn5+ EVs in triggering inflammation, offering a novel mechanism involved and presenting a possible therapeutic avenue for DR.

## 1. Introduction

Diabetic retinopathy (DR), a common complication of diabetes (db), is characterized by several features including the loss of retinal pericytes, abnormal angiogenesis, and inflammation [1,2]. It is a significant public health concern and a leading cause of irreversible blindness in the working-age population worldwide [3]. The precise molecular mechanisms driving the pathogenesis of DR remain elusive. Dysregulation of extracellular vesicles (EVs) has been reported to play a key role in the development of vascular damage and the progression of DR [4,5]. Previous studies found that plasma EVs from DR patients could induce retinopathy features in retinal microvasculature models [6]. There is insufficient knowledge about the presence, source, and function of retinal EVs in DR.

EVs are signaling particles without replicative capacity, released by diverse types of cells to the biological fluids to facilitate intercellular communication [7]. According to the International Society for Extracellular Vesicles, EVs can be classified as small EVs (diameter < 200 nm) and medium/large EVs (diameter > 200 nm) [8]. EVs can enter cells by fusing with the membrane without the need for a specific receptor [9]. The role of EVs in cellular damage and dysfunction, particularly in the context of DR, has garnered increasing attention [4]. EVs have been implicated in various physiological and pathological processes, including those associated with DR [10,11,12]. Despite this recognition, the specific characteristics and functions of EVs in the context of DR remain poorly understood.

Vitreous is the preferred biofluid for postmortem biochemical investigation of ocular pathologies due to its large volume and ease of access [13]. Since the vitreous directly interfaces with the retina, changes in its content likely reflect retinal injury and vascular inflammation in db patients [14]. Vitreous EVs are increasingly recognized for their role in the pathophysiology of diabetes (db)-related ocular complications. In db, the altered metabolic environment might also affect the composition and function of vitreous EVs, contributing to inflammation, oxidative stress, and vascular dysfunction in the retina [15]. While reports exist on EVs derived from cell culture systems and retinal tissues in relation to ocular diseases [5], our understanding of EVs from db vitreous—specifically concerning their potential sources in vivo—uptake by inflammatory cells and their role in triggering inflammation remains limited.

Our study addresses the existing gap in knowledge by investigating the characteristics of type 2 db vitreous EVs and exploring their pro-inflammatory properties. We aimed to provide a comprehensive understanding of the heterogeneity of vitreous EVs in both type 2 db and non-db conditions. This includes elucidating their sub-populations and size distribution through advanced techniques such as nanoparticle tracking analysis (NTA) and transmission electron microscopy (TEM). By examining the cytokine levels and through proteomic and bioinformatics analysis, we sought to delineate the influence of db vitreous EVs on macrophage activation, offering valuable insights into the inflammatory pathways occurring in the db retina. The increased presence of Cldn5 in the db vitreous EVs with the ability of triggering macrophage activation suggest endothelial cells are their potential origin, and the potential involvement of Cldn5+ EVs in retinal injury and inflammation in db patients.

## 2. Materials and Methods

### 2.1. Patient Characteristics

Twelve vitreous samples were obtained for this study from the National Disease Research Interchange (Philadelphia, PA). These samples included 6 from individuals with type 2 db mellitus (with a disease duration of around 15 years) and 6 from non-db individuals. The baseline characteristics of the study subjects showed that the average age between the db and non-db groups was comparable, at 68.35 ± 8.827 years and 70 ± 10.74 years, respectively. Other baseline characteristics, including race, gender, presence of health complications such as heart attacks, and the use of tobacco or alcohol, were similarly matched. None of these patients received any form of intravitreal treatment. The analyses revealed no statistically significant differences in these variables between the two groups (See Results section). The db group had been living with the disease for an average of 15 years.

### 2.2. Isolation of Human Vitreous EVs

EVs were isolated from the human vitreous humors using a sequential centrifugation protocol with slight modifications according to the established protocol [16]. In brief, human vitreous samples were centrifuged at 500× *g* for 5 min to remove cells. The supernatant was centrifuged at 2000× *g* for 20 min to eliminate apoptotic bodies and cell debris. The resulting supernatants were mixed with an equal volume of a 2× PEG (16% PEG with 1 M NaCl) solution and were incubated at 4 °C overnight, followed by centrifugation at 3000× *g* for 1 h to pellet total EVs. The resulting supernatants were filtered using a 0.22-μm filter and ultracentrifuged at 120,000× *g* for 1 h at 4 °C to pellet EVs. To wash EVs, PBS was added and EVs were ultracentrifuged at 120,000× *g* for 1 h at 4 °C. Isolated EVs were stored at −80 °C immediately after isolation until further analysis or further characterized using nanoparticle tracking analysis (NTA), imaged with transmission electron microscope (TEM), immuno-EM, and Western blotting, as previously described [17].

### 2.3. Nanoparticle Tracking Analysis

NTA was used to measure the size and concentration of EVs [18]. NTA of human vitreous EVs was performed using the ZetaView PMX 120 (Particle Metrix, Meerbusch, Germany) and its corresponding software (ZetaView 8.02.28) according to the established protocol [19]. Briefly, all samples were diluted using 1×PBS, and the instrument measured each sample at 11 different positions with two cycles of readings at each position. After automated analysis, including removal of outlier positions, the mean, median, and mode sizes, along with sample concentration, were calculated using the optimized machine software. Instrument pre-acquisition parameters were set to a temperature of 23 °C, sensitivity of 85, frame rate of 30 fps, shutter speed of 100, and laser pulse duration equal to shutter duration. Post-acquisition parameters were set to a minimum brightness of 25, maximum size of 200 pixels, and minimum size of 5 pixels. Calibration using polystyrene particles of known average size preceded sample readings. Automated quality control measures, including cell quality check, instrument alignment, focus, and recording of temperature, conductivity, electrical field, and drift measurements, were performed. The mode (diameter) was selected for size measurement. Resuspension volumes and dilution factors were considered for accurate conversion of concentration to an absolute number of particles. Quadratic interpolation was applied to generate the number of particles per particle size curves. Zeta potential measurements were conducted at 23 °C using ZetaView PMX 120 with 0.05× diluted PBS, following the same protocol as for size and concentration measurements. Measurements were performed on experimental triplicates of each starting volume of EVs. Data analysis was performed using ZetaView 8.02.28 software.

### 2.4. TEM and Immunogold Labelling Studies

For TEM, freshly isolated EV suspensions, diluted at a ratio of 1:10, were fixed in 4% paraformaldehyde for 1 h. A volume of approximately 5 μL from different samples was applied to copper mesh Formvar-coated carbon-stabilized grids, allowed to adsorb for 4–5 min, and then wicked off with filter paper. Negative staining was carried out with 1% aqueous uranyl acetate (5 μL) applied for 30 s, followed by removal with Whatman filter paper. Grids were thoroughly dried before TEM examination using a JEM 1230 (JEOL USA Inc., Peabody, MA, USA). The preparation and imaging of EV suspensions were conducted at the Electron Microscopy and Histology Core Laboratory at Augusta University as standardized in the laboratory [16].

EVs were immunolabeled using the anti-Claudin5 antibody (Invitrogen, Waltham, MA, USA) and mouse IgG1 control (Proteintech, Rosemont, IL, USA). EV samples were diluted at a ratio of 1:10 and then fixed overnight in 4% paraformaldehyde, diluted in 0.1 M cacodylate buffer (pH 7.4). Fixed exosome samples (20 μL) were applied to carbon–Formvar-coated 200 mesh nickel grids and allowed to dry for 30 min before removing excess exosomes using Whatman filter paper. The grids were then floated with the sample side down onto a 20 μL drop of 1.0 M ammonium chloride for 30 min to quench aldehyde groups from the fixation step, followed by floating on drops of blocking buffer (0.4% BSA in 1× PBS) for 2 h. After rinsing three times (5 min each) with 1× PBS, the grids were incubated with either blocking buffer only (negative control) or primary antibodies (Cldn5 or IgG1 control) diluted with blocking buffer (1:100) for 1 h. Subsequent steps included rinsing the grids with deionized water (3 times for 5 min each) and 1× PBS after the incubation, floating the grids on drops of 1.4 nm anti-mouse nanogold (Nanoprobes, Inc., Yaphank, NY, USA) diluted 1:1000 in blocking buffer for 1 h, enhancing the grids using HQ Silver (gold enhancement reagent, Nanoprobes, Inc.) for 1 min, followed by rinsing in ice-cold deionized water. As a final step, negative staining in 2% aqueous uranyl acetate was performed, and samples were wicked dry and allowed to air dry. TEM imaging was conducted using a JEM 1230 (JEOL USA Inc., Peabody, MA, USA) at the Electron Microscopy and Histology Core Laboratory at Augusta University.

### 2.5. Western Blot Analysis

Western blot analyses were performed according to the previous methods [16]. The EVs were processed in 1× RIPA lysis buffer (Millipore, Billerica, MA, USA) supplemented with 1× protease and phosphatase inhibitors (Thermo Scientific, Waltham, MA, USA) and then centrifuged at 12,000× *g* for 15 min to obtain protein extracts. The protein concentration was determined using a Pierce BCA protein assay kit (Thermo Scientific, Waltham, MA, USA). Subsequently, EV proteins were separated on SDS-PAGE, transferred to nitrocellulose membranes (Millipore, Billerica, MA, USA), and blocked in 5% non-fat dry milk in 1× Tris-buffered saline (TBS) with 0.1% Tween-20 (TBST) for 1 h. Membranes were incubated overnight at 4 °C with the corresponding primary antibodies (Table 1). The following day, membranes underwent washing with 1× TBST (3 times) and were incubated with the appropriate secondary antibodies (anti-rabbit or anti-mouse HRP-conjugated secondary antibody, Table 1) for 2 h at room temperature. Immunoreactive proteins were detected on the membranes using an enhanced chemiluminescence (ECL) system (Thermo Scientific, Waltham, MA, USA) and the ChemiDoc Imaging System (Bio-Rad, Hercules, CA, USA). Densitometry analysis was performed using ImageJ software (Version 1.53m), and the data were normalized to β-actin as the loading control.

### 2.6. Cell Culture

Human THP-1 monocytes (ATCC #TIB-202) were used for this study. THP-1 cells were obtained from ATCC (Product No. TIB-202) and maintained in RPMI-1640 with 10% fetal bovine serum, 1% penicillin–streptomycin, and 2 mM L-glutamine. THP-1 cells were differentiated into macrophage-like cells for all the experiments by 150 nM and 10 nM PMA treatment for 24 h according to our previous reports [20]. All the cells were maintained at 37 °C in a humidified atmosphere of 5% CO_2_/95% air. Cell density was determined using a hemocytometer, and cells were seeded at 1 × 10^5^ to 1 × 10^6^ cells/mL for experiments.

### 2.7. Uptake of Human Vitreous EVs with Fluorescence Labeling by THP-1 Macrophages

The evaluation of intracellular uptake of human vitreous EVs at concentration of 10 ug/mL by THP-1 macrophages was assessed using an ExoGlow™ Membrane EV Labeling Kit (System Biosciences, Palo Alto, CA, USA) by confocal microscopy at 24 h For the uptake of human vitreous EVs with fluorescence labeling by THP-1 macrophages, a labeling protocol was executed according to the product manual. Briefly, 2 μL of labeling dye was added in 12 μL of reaction buffer and thoroughly mixed until complete dissolution, resulting in the formation of the labeling reaction buffer. Subsequently, 50–100 μg of vitreous EVs were introduced into the labeling reaction buffer, and the sample was well mixed before undergoing a 30 min incubation at room temperature, with a precautionary measure to shield the tubes from light. This labeled EV preparation is then used for the introduction to THP-1 macrophages for subsequent uptake studies. The cells were then observed and photographed using a fluorescence microscope. 

### 2.8. RNA Isolation and Quantitative RT-PCR 

The impact of EVs on macrophage (THP-1) activation was assessed by cytokine levels using RT-qPCR. The differentiated THP-1 macrophages with EV treatment for 6 h (EV 10 ug/mL, 5 samples per group). Manipulations to perform include the treatment of differentiated THP-1 macrophages with Cldn5+ EVs. Control groups will be macrophages treated with PBS. We further evaluate the impact of Cldn5+ EVs by performing pro-inflammatory cytokine levels using RT-qPCR (using the cell extracts) as described [21]. In brief, the total RNA from macrophage (THP-1) was extracted using an RNeasy mini kit (Qiagen). The concentration of RNA was measured using a Nanodrop Lite Spectrophotometer (Thermo Fisher Scientific, Waltham, MA, USA). Around 500 ng of total RNA was used for cDNA synthesis using a High-Capacity cDNA Reverse Transcription Kit (Applied Biosystems, Waltham, MA, USA). Quantitative PCR was carried out by StepOnePlus™ Real-Time PCR System (Applied Biosystems) using a Power SYBR Green Master Mix (Applied Biosystems). The sequences of primers used in this study are listed in Table 2. Data were normalized to beta-actin (β-actin), and the fold change between levels of different transcripts was calculated by the ΔΔCT method.

### 2.9. Proteomic Analysis

Liquid chromatography–tandem mass spectrometry (LC-MS)-based proteomic analysis was performed at Augusta University’s Proteomics Core facility. EVs from 12 vitreous humor samples, 6 db, and 6 non-db subjects were isolated and subjected to protein quantification assessment using the BCA protein assay kit (Cat#23225, Thermo Scientific, Waltham, MA, USA). Roughly 50–100 μg of total protein was used for LC-MS analysis. All the raw data is presented in Table S1.

### 2.10. Bioinformatics Analysis

The raw mass spectrometry data were managed using Proteome Discoverer software (version 1.4, Thermo Scientific). Trimmed Mean of M-values (TMM) was used to transform and normalize the data with the R library. The abundance changes in protein level between db EVs and non-db EVs in vitreous samples after normalization were determined via Student’s *t*-test with a *p*-value less than 0.05 and a fold change of more than 2. Peptide spectral match (PSM) totals were calculated to evaluate the relative abundance of proteins in EVs of the vitreous samples. Analysis of protein distribution, such as principal component analysis (PCA), volcano plot, and heatmap analysis, was performed using R version 4.3.2. Detailed, functional kinase enrichment and pathway analyses were performed on proteins that were differentially expressed in EVs of the vitreous humor of db and non-db subjects employing Enricher data analysis and SRplot [22,23,24]. The pathway analysis of these proteins was conducted on multiple databases such as the Kyoto Encyclopedia of Genes and Genomes (KEGG), Wiki, and Reactome. The GO database was used to scrutinize the cellular components, biological processes, and molecular functions of identified proteins. The STRING database was utilized to demonstrate the protein-protein interactions [25].

### 2.11. Statistical Analysis

The data are expressed as mean ± standard deviation (SD). Statistical analysis was conducted using GraphPad Prism 9 (GraphPad Software Inc., La Jolla, CA, USA). One-way analysis of variance (ANOVA) followed by the Tukey test for multiple comparisons was employed for data analysis. In instances of single comparisons between means of two groups, Student’s *t*-test was utilized. We also conducted the comparison of clinical parameters, including the duration of diabetes listed in the demographic table, using a non-parametric, two-tailed Mann–Whitney U test, which yielded the same level of statistical significance. To mitigate selection bias, analyses were carried out in a blinded manner and quantified by three independent observers using ImageJ software. Significance was established at a *p* value less than 0.05.

## 3. Results

### 3.1. Characterization of Extracellular Vesicles Isolated from Human Vitreous Samples

**Figure 1:**
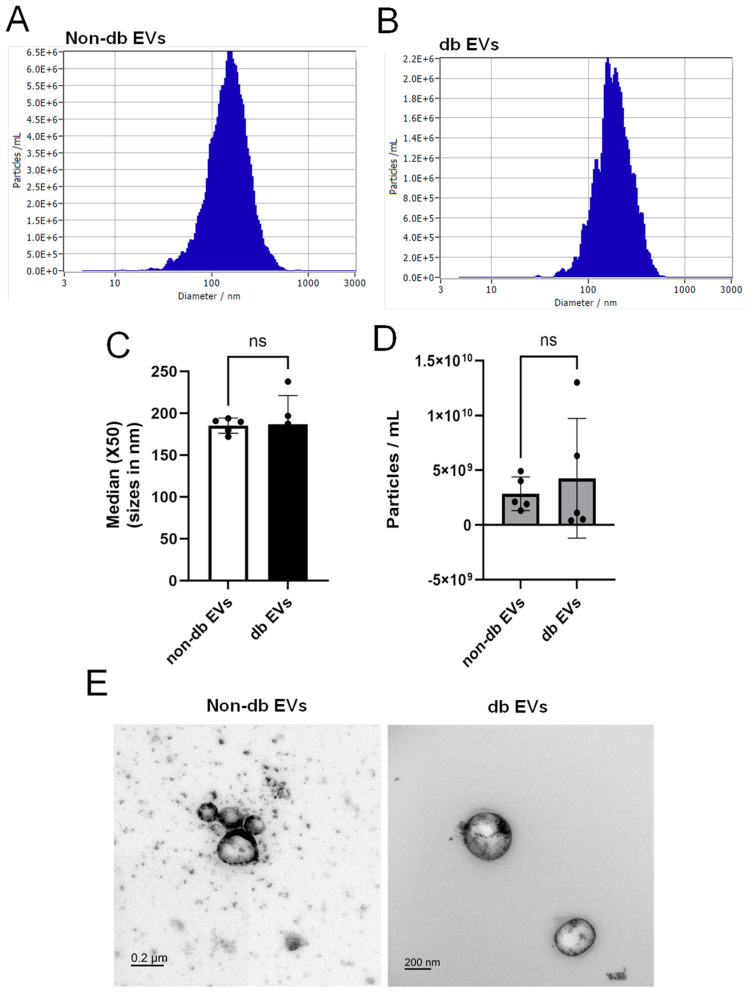
Characterization of human vitreous EVs. (**A**,**B**) Size distribution of EVs purified from non-db and db vitreous. (**C**) Average diameters of non-db and db vitreous EVs. (**D**) Average particle numbers in non-db and db vitreous EV preparations. Size distribution was averaged for each group. (**E**) TEM images of isolated EVs from non-db and db vitreous EVs revealing non-aggregated EVs. ns, Not Significant. Scale bar = 200 nm.

We isolated EVs from human vitreous samples (Table 3) and characterized them by NTA to determine whether there were differences in vitreous EV concentration and size distribution between db and non-db samples. NTA results revealed that the average size of control and db vitreous EVs were 185.2 ± 9.084 nm and 186.9 ± 34.36 nm, respectively (Figure 1A–C); the average particle numbers of non-db EVs and db EVs were 2.84 × 10^9^ and 4.26 × 10^9^, respectively (Figure 1D). Although the size and concentration of vitreous EVs were slightly larger in the db group compared to the non-db group, these differences were not statistically significant (Figure 1C,D). Transmission electron microscopy (TEM) revealed the morphology of vitreous EVs showing non-aggregated EVs in both groups with a heterogeneous size range around 200 nm (Figure 1E). 

### 3.2. Vitreous EVs Express Specific Markers and Endothelial Junctional Protein Claudin-5

**Figure 2:**
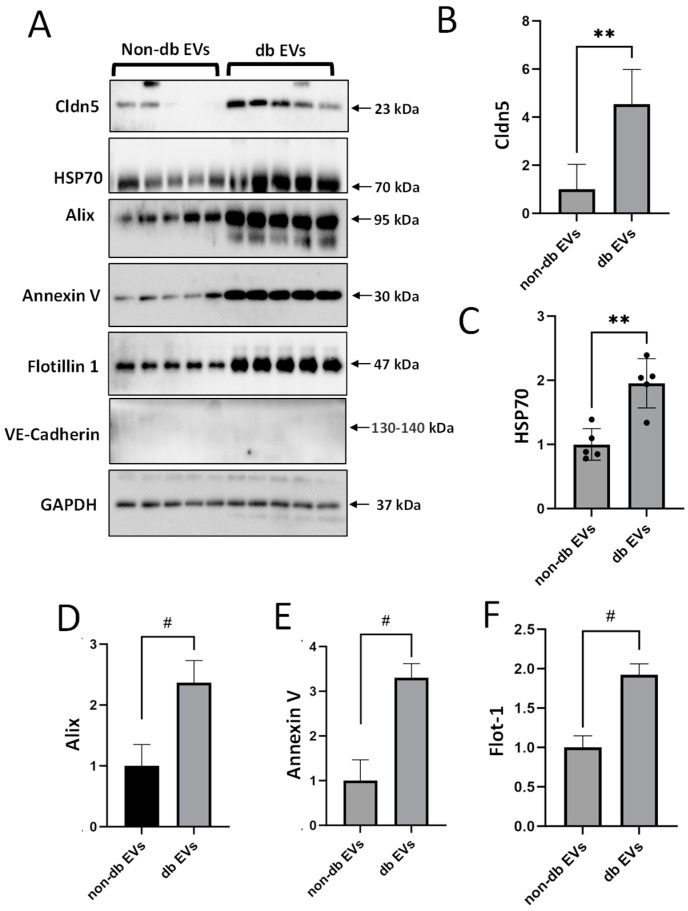
Db vitreous EVs exhibit increased expression of EV markers and Cldn5. (**A**) Representative Western blot images of known EV markers (Alix, annexin V, HSP70, and flotillin 1) in non-db and db vitreous EVs (EVs, *n* = 5, *p* < 0.01). (**B**) Bar graph showing increased expression of endothelial junctional protein, Cldn5, in db compared to non-db vitreous EVs (*n* = 5, ** *p* < 0.01; # *p* < 0.001). (**C**–**F**) Bar graphs showing the presence of EV markers in both groups.

**Figure 3:**
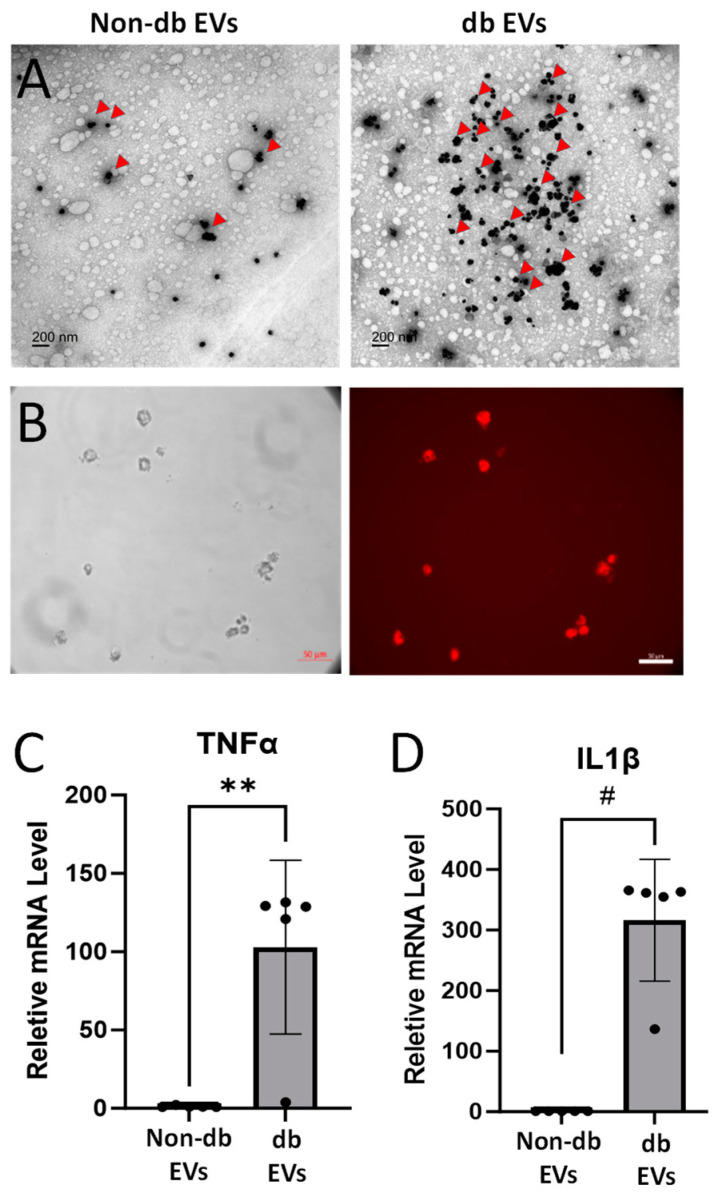
Macrophages uptake db EVs to induce cytokine synthesis. (**A**) Immunogold labeling confirms the increased presence of Cldn5 in db vitreous EVs (red arrowhead). This confirms the significant presence of Cldn5 in the vitreous EVs of individuals with diabetes. (**B**) Fluorescence microscopy images showing the uptake of ExoGlow membrane-labeled human db vitreous EVs (10 ug/mL) by THP-1 macrophages after 24 h (40×). (**C**,**D**) qRT-PCR analysis of THP-1 macrophage post db vitreous EV treatment for 6 h (EV 10 ug/mL) demonstrating changes in the mRNA levels of pro-inflammatory cytokines TNFα and IL-1β, respectively (*n* = 5). Data are presented as mean ± SD. ** *p* < 0.01; # *p* < 0.001.

Western blot analyses detected EV markers (Alix, Annexin V, HSP70, and Flotillin 1) in both db and non-db vitreous EVs (*n* = 5; *p* < 0.01) (Figure 2A–F) with significantly increased expression in the db EV preparations. The potential significance of Claudin-5 (Cldn5), a predominant endothelial tight junction protein in the pathogenesis of retinopathies, is underscored by our recent findings in vitro [26] and in an experimental model of OIR [21]. To determine the presence of Cldn5 in db EVs and determine their endothelial origin, we evaluated the Cldn5 expression in the db EVs. Western blot analyses showed an upregulation of Cldn5 in db EV vitreous samples (*n* = 5; *p* < 0.01), while VE-cadherin was undetected (Figure 2A,B). Immunogold labeling also demonstrated that Cldn5 was abundantly expressed in db EVs (red arrowhead) compared to non-db EV preparations (Figure 3A).

### 3.3. Db Vitreous EVs Induced THP-1 Macrophage Activation and Cytokine Production 

Fluorescence microscopy showed that the db vitreous EVs were taken up by differentiated THP-1 macrophages with almost all the cytoplasmic area positive for the EVs (red). Figure 3B shows fluorescence images of THP-1 cells 24 h after EV addition at the dosage of 10 µg/mL. The intracellular uptake of db EVs by the macrophages prompted further investigation into its potential role in the inflammatory response. The qRT-PCR analysis showed that treatment of THP-1 cells with db EVs significantly increased mRNA levels of pro-inflammatory cytokines (TNFα and IL-1β) compared to non-db EVs (*n* = 5; *p* < 0.01), suggesting that EVs from db vitreous may promote inflammation (Figure 3C,D).

### 3.4. Proteomic Profiling of EVs from db Vitreous Samples

**Figure 4:**
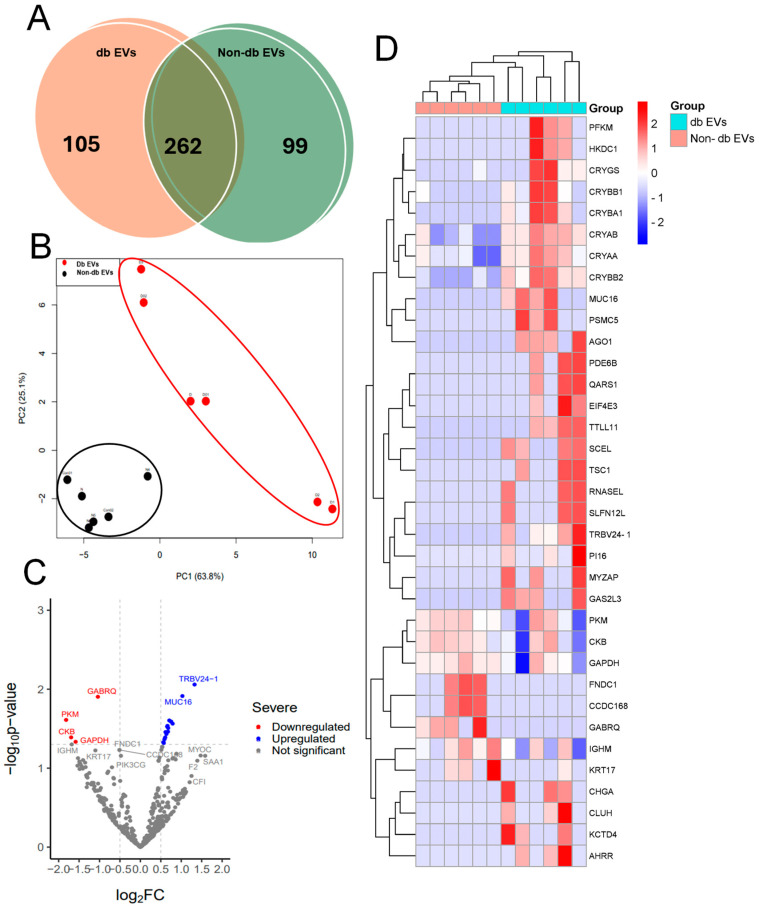
Proteins with differential expression in the db vitreous EVs. (**A**) Venn diagram showing the detailed counts of identified proteins in db and non-db EVs. (**B**) Principal component analysis (PCA) indicating the two groups were separated. (**C**) The volcano plot of db versus non-db vitreous EVs demonstrating the proteins that are significantly altered among the groups. (**D**) The heatmap showing differentially expressed proteins in db compared to non-db vitreous EVs (*n* = 6).

Next, we performed mass spectrometry analysis to identify the differences between db EVs and non-db EVs. We observed a total of 3961 unique proteins in all groups. Of these identified proteins, only 466 were present in half of all the examined samples, with 105 and 99 proteins detected in db and non-db vitreous EVs, respectively. The top 10 most abundant proteins in either of the vitreous EV groups are shown in Table 4. Furthermore, 262 proteins were commonly expressed in both groups (Figure 4A). The principal component analysis (PCA) revealed that the two groups were distinctly separated according to their protein expression changes (Figure 4B). To further explore significant differences in protein expression levels in db EVs compared to non-db EVs, we identified around 27 proteins that are differentially expressed, with 23 increased and 4 decreased in db EVs, as shown in the volcano plot and the heatmap (Figure 4C,D). The top proteins found to be significantly higher or lower in db versus non-db vitreous EVs are listed in Table 5 and Table 6.

### 3.5. Pathways Enrichment Analysis of the Differentially Expressed Proteins Indicate Modulation of Metabolic and Inflammatory Pathways in db EVs

**Figure 5:**
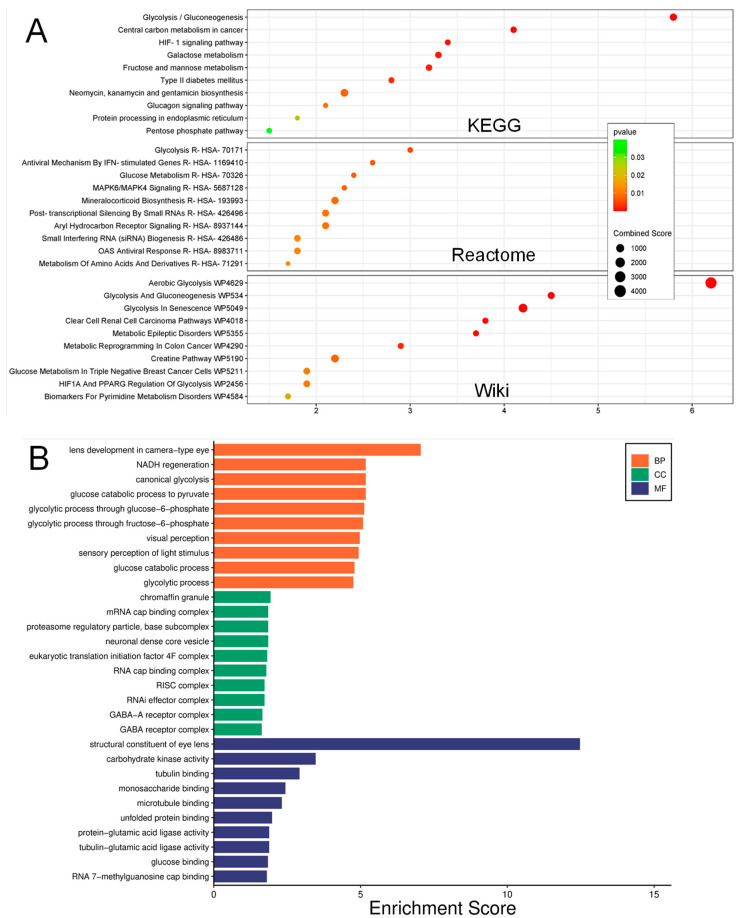
Gene enrichment analysis: (**A**) KEGG, Reactome, and Wiki pathway analysis of db vitreous EVs showing involvement of several inflammatory pathways. (**B**) GO analysis showing top enriched ontologies in the biological processes, cellular components, and molecular functions of deferentially expressed proteins in db vitreous EVs compared to non-db EV control.

Multiple pathway enrichment analysis databases were used to comprehensively explore pathways linked with the identified differentially expressed proteins. We observed the presence of more than 30 dysregulated pathways in the db vitreous EVs. In KEGG analysis, we found that altered proteins were significantly associated with several inflammatory pathways, such as the HIF-1 and protein processing in the endoplasmic reticulum (Figure 5A). Although the analysis in Reactome mainly revealed changes in metabolic pathways, mitogen-activated protein kinase (MAPK), one of the main inflammatory pathways, was significantly found to be modulated in EVs from db vitreous (Figure 5A). Similarly, the Wiki pathway platform revealed the involvement of HIF1A pathways in the db-vitreous EVs (Figure 5A).

**Figure 6:**
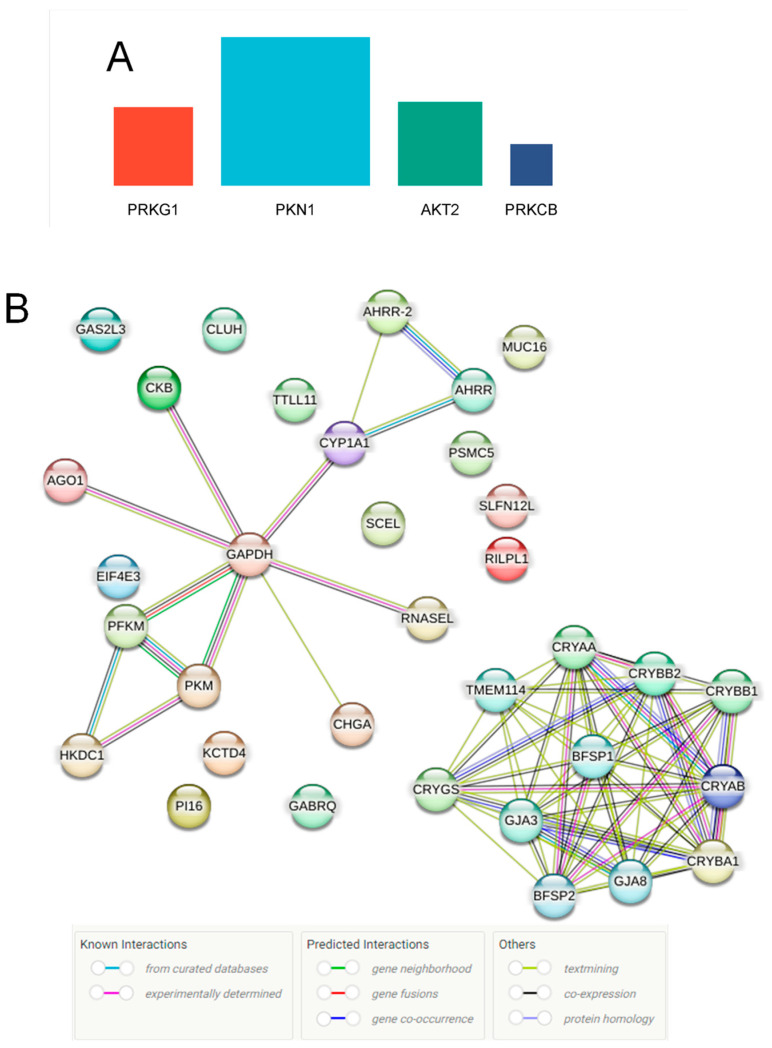
(**A**) Kinase enrichment analysis showing four significant protein kinases of differentially expressed protein. (**B**) Protein–protein interaction (PPI) network demonstrating 27 differentially regulated proteins generated by the STRING database.

Using the Gene Ontology (GO) database, we evaluated the biological roles, molecular functions, and cellular components associated with differentially expressed proteins in db vitreous EVs (Figure 5B). In biological function analysis, we observed that these altered proteins were significantly associated with lens development, glycolytic process, visual perception, sensory perception of lighting stimulus, and NADH regeneration (Figure 5B). When we investigated the cellular components of the changed proteins in vitreous db samples, we found that chromaffin granule, mRNA cap-binding complex, proteasome regulatory particle, neuronal dense core vesicle, and GABA receptor complex were pertinent to differentially expressed proteins (Figure 5B). Lastly, the results of the top molecular functions analysis showed the involvement of structural constituents of the eye lens, carbohydrate kinase activity, tubulin binding, microtubule binding, and glucose binding. Considering the importance of kinase enrichment analysis, we subjected the significantly changed proteins to the kinase library through the Enricher platform. Our findings revealed that only four statistically significant enriched kinases were detected, including PRKG1, PKN1, AKT2, and PRKCB (Figure 6A). To identify the interactions between the differentially expressed proteins, including all the upregulated and downregulated proteins, a network demonstrating protein–protein interaction was constructed using the STRING database. Our analysis showed that several interactions were identified among the proteins, along with others that had no interactions (Figure 6B). Further, we examined the altered proteins in terms of their contribution to diseases using the DISEASES database via Enricher. This analysis resulted in identifying several relevant diseases to the eye, with the most significant diseases being cataracts and blindness (Table 7).

## 4. Discussion

DR is a major cause of blindness in the working-age population globally, marked by intricate pathological processes such as retinal vascular damage, inflammation, and abnormal blood vessel growth [27]. Despite extensive research efforts, the exact molecular mechanisms driving DR are still not fully understood, and the current treatments available are both limited in their effectiveness and accessibility [28]. Emerging evidence suggests that EVs play a critical role in mediating intercellular communication and may contribute to the pathogenesis of DR by transporting bioactive molecules that modulate inflammation and vascular dysfunction [4,5,6,10]. Although our recent study reported pro-inflammatory and metabolic pathway markers in db vitreous versus non-db vitreous proteomic analysis [14], there is a significant knowledge gap regarding the specific characteristics and functions of EVs in the vitreous of db patients. This study aimed to address this gap by comprehensively characterizing EVs, specifically from type 2 db and non-dB vitreous samples, investigating their potential pro-inflammatory properties, and elucidating their role in the pathophysiology of DR. By gaining a deeper understanding of the molecular composition and effects of db vitreous EVs, we hope to uncover novel biomarkers and therapeutic targets for the prevention and treatment of DR.

Our study revealed significant differences in the EVs isolated from the vitreous humor of type 2 db and non-db individuals, suggesting a potential role of EVs in the pathogenesis of DR. This observation aligns with another recent report although the latter study recruited non-db subjects diagnosed with idiopathic macular epiretinal membrane and idiopathic macular hole needing PPV surgery as the control group against the healthy, non-db subjects in our analysis [15]. NTA and TEM analysis demonstrated that the size and concentration of vitreous EVs were comparable between db and non-db samples, although there was a trend toward higher concentration and size in db versus non-db vitreous.

Consistent with previous reports [29], we found that vitreous EVs from db individuals exhibit distinct molecular signatures compared to those from non-db controls, with an upregulation of standard EV markers such as Alix, annexin V, HSP70, and flotillin 1, and a notable increase in Cldn5, an endothelial tight junction protein. In a recent study, we reported that Cldn5 is upregulated in human retinal endothelial cells upon treatment with high glucose and advanced glycation end-products, and in an oxygen-induced retinopathy model in mice. In light of this, an upregulation of Cldn5 in db vitreous EVs suggests the potential endothelial origin of these EVs, implicating them in endothelial dysfunction and increased vascular permeability observed in DR. This finding is consistent with earlier research indicating that EVs derived from endothelial cells under db conditions can carry markers of endothelial injury and contribute to vascular inflammation and permeability [30]. Moreover, the absence of VE-cadherin in these EVs reinforces the selective packaging of specific proteins under db conditions [31].

While most studies on EVs isolated from liquid biopsies are focused on morphological and molecular characterization [32], fewer studies have been conducted investigating their effects on cell types. In particular, the effect of EVs from the human vitreous on inflammatory cells has never been conducted. Hence, we delved into the biological effects of db EVs, which revealed that the db vitreous EVs were readily taken up by differentiated THP-1 macrophages. Furthermore, treatment of THP-1 cells with db vitreous EVs leads to a significant increase in the mRNA expression of pro-inflammatory cytokines TNFα and IL-1β, indicating that db EVs may promote an inflammatory response in the retinal microenvironment. Previous studies have shown that EVs can act as carriers of pro-inflammatory mediators and facilitate the transfer of inflammatory signals between cells, thereby exacerbating retinal inflammation in db [4,33]. The ability of EVs to induce macrophage activation [34] further underscores their role in modulating immune responses in the injured retina [35], highlighting the potential contribution of EVs in inducing inflammation to retinal microvascular damage in DR [36].

The repertoire of proteins present in type 2 db vitreous EVs is currently unknown. Our proteomic profiling identified a total of 3961 unique proteins in db and non-db vitreous EVs, with 105 and 99 proteins specifically detected in db and non-db vitreous EVs, respectively. Among these, we identified around 27 proteins that are differentially expressed, with 23 increased and 4 decreased in db EVs, compared to non-db controls. Whereas the most significantly upregulated proteins in db vitreous EVs include beta-crystallin B2, alpha-crystallin B chain, alpha-crystallin A chain, beta-crystallin B1, beta-crystallin A3, gamma-crystallin S, T cell receptor beta variable 24-1, mucin-16, sciellin, and hexokinase, proteins that are downregulated include glyceraldehyde-3-phosphate dehydrogenase, creatine kinase B-type, pyruvate kinase PKM, and gamma-aminobutyric acid receptor subunit theta. Whereas mucin-16 (MUC16) has been implicated in keratoconjunctivitis [37], crystallins have been implicated in micro- and macro-glial activation and neuroinflammation [38,39,40]. Whereas crystallins could improve neuronal cell survival through Müller cell secretion [41], glycation and phosphorylation of crystallins in db/chronic hyperglycemia conditions could alter their structure and function resulting in neuronal injury [42,43,44], indicating a paradoxical and context-specific effect of these proteins in the retina.

Differential protein expression analysis using various databases (KEGG, Reactome, Wiki Pathways) highlighted the enrichment of pathways related to inflammation and metabolism in db EVs, including the HIF-1 and MAPK signaling pathways. This observation is consistent with the literature reporting EVs from db kidneys [45,46] and db retinas [47] showing differential protein expression linked to metabolic and inflammatory processes. GO analysis further associated the differentially expressed proteins with biological processes such as lens development, glycolytic process, visual perception, and NADH regeneration. The altered proteins were also linked to specific cellular components and molecular functions pertinent to DR pathogenesis. The enrichment of proteins involved in pathways such as HIF-1 and MAPK suggests that EVs play a role in hypoxia and stress responses in the db retina, aligning with previous research on the role of these pathways in DR progression [48,49]. Furthermore, our study identified specific kinases such as PRKG1, PKN1, AKT2, and PRKCB associated with the altered proteins in db EVs, suggesting their involvement in the modulation of EV functions and contributions to DR pathophysiology. This is supported by earlier research demonstrating the importance of kinase signaling in EV biogenesis and cargo selection [50], as well as in mediating cellular responses to db conditions.

In conclusion, our study offers significant insights into the altered characteristics and pro-inflammatory properties of vitreous EVs in individuals with type 2 db, underlining their potential role in the pathogenesis of DR. We observed notable differences in the molecular signatures of EVs between db and non-db vitreous samples, including an upregulation of specific EV markers and endothelial proteins such as Cldn5. Although additional studies will be necessary to prove this point, the gene enrichment analysis suggests that EVs contribute to endothelial dysfunction and vascular permeability, key features of DR. Our proteomic analysis identified a plethora of differentially expressed proteins associated with inflammation and metabolic pathways, highlighting the enrichment of pathways like HIF-1 and MAPK signaling in db EVs. Furthermore, the ability of db vitreous EVs to induce pro-inflammatory cytokine expression in macrophages points to their role in exacerbating retinal inflammation. Despite these new findings, this study comes with several limitations. The relatively small sample size limited our ability to fully analyze patient characteristics, such as any potential differences in associated risk factors, warranting larger sample size analysis in the future to detect significant differences. The vitreous were collected postmortem, and some patients had comorbidities such as heart disorders, which could have influenced the gene ontology analysis of molecular functions and biological processes, potentially leading to findings not directly relevant to db retinal complications. Furthermore, although some patients had a history of db for approximately 20 years, the data regarding development of DR was unavailable; however, it was anticipated based on the duration of db in these patients. The study included only type 2 db patients, excluding type 1 db from our analysis, which is a strength (the results could be specific to type 2 db) as well as a weakness (the data for type 1 db could be different). The exact reason for the increased expression of EV markers and the endothelial marker Cldn5 in db EVs compared to control EVs is also unclear from our study, despite the relatively similar particle size and number between the two groups. Finally, the exact role of each differentially expressed protein, whether upregulated or downregulated in the context of db, requires further experimental investigation to understand their significance and contribution to the pathogenesis of eye diseases. Nevertheless, our comprehensive characterization underscores the significance of vitreous EVs as mediators of intercellular communication and potential biomarkers for DR. This study emphasizes the necessity for further research to elucidate the molecular mechanisms by which EVs contribute to DR progression, potentially paving the way for novel therapeutic strategies targeting these vesicles.

## Figures and Tables

**Table 1:** List of antibodies used in this study.

Antibody	Catalog #	Source	Dilution	Experiment
Claudin 5	35-2500	Invitrogen, Waltham, MA, USA	1:1000	Western blot
GAPDH (14C10)	2118	Cell signaling, Danvers, MA, USA	1:1000	Western blot
HSP70	4876	Cell signaling	1:1000	Western blot
Alix (3A9)	2171	Cell signaling	1:500	Western blot
Annexin V	8555	Cell signaling	1:500	Western blot
VE-cadherin (D87F2)	2500	Cell signaling	1:500	Western blot
Anti-flotillin 1	ab133497	Abcam, Waltham, MA, USA	1:500	Western blot
Goat anti-rabbit IgG (H + L)–HRP conjugate	1706515	Biorad, Hercules, CA, USA	1:5000	Western blot
Goat anti-mouse IgG (H + L)–HRP conjugate	1721011	Biorad	1:5000	Western blot
Claudin 5	35-2500	Invitrogen	1:100	Immunogold labelling
IgG1 isotype control	66360-1-Ig	Proteintech, Rosemont, IL, USA	1:100	Immunogold labelling

**Table 2:** Primer sequences used in this study.

Gene Name	Forward Primer	Reverse Primer
TNFα	GGTCCCCAAAGGGATGAGAA	TGAGGGTCTGGGCCATAGAA
IL1β	CCAAGCAACGACAAAATACC	GTTGAAGACAAACCGTTTTTCC
Beta-actin	TCCCTGGAGAAGAGCTACGA	AGCACTGTGTTGGCGTACAG

**Table 3:** Demographics of the diabetic patients and non-diabetic controls.

	db Patients	Non-db Individuals	*p*-Value
*n*	6	6	
Race			
Caucasian	4 (66.7)	5 (83.3)	NS
Black	2 (33.3)	1 (16.7)	NS
Age (years)	68.35 ± 8.827	70 ± 10.74	NS
Gender, n (%)			
Male	3 (50)	3 (50)	NS
Female	3 (50)	3 (50)	NS
BMI (kg/m^2^)	32.45 ± 7.84	30.77 ± 6.29	NS
Weight (lbs)	193.8 ± 64.15	201.3 ± 57.21	NS
Height (inches)	66.61 ± 5.33	66.70 ± 4.56	NS
Tobacco use, n (%)	5 (83.3)	6 (100)	NS
Alcohol use, n (%)	4 (66.7)	3 (50)	NS
Duration of diabetes (years)	15.41 ± 9.72	0	<0.0001
Complications, n (%)			
Heart attack/failure	5 (83.3)	4 (66.7)	NS
Medication, n (%)			
Hypoglycemic medications	6 (100)	0 (0)	<0.0001
Heart medications	6 (100)	5 (83.3)	NS

Data are shown as mean ± SD. *p*-value, diabetic versus non-diabetic controls. Abbreviations: BMI—body mass index; NS—not significant; SD—standard deviation.

**Table 4:** Ten most abundant proteins present in vitreous EVs.

#	Accession Number	Gene Symbol	Gene Name	Σ# PSMs		ΔPSM	Molecular Weight [kDa]
				Diabetic	Normal		
1	P02768	*ALB*	Albumin	30,007	24564	5443	69.3
2	P01834	*IGKC*	Immunoglobulin kappa constant	1816	1546	270	11.8
3	P02787	*TRFE*	Serotransferrin	9026	6348	2678	77.0
4	P10745	*RET3*	Retinol-binding protein	1736	1266	470	135.3
5	P01009	*α1AT*	Alpha-1-antitrypsin	1768	1087	681	46.7
6	P01024	*CO3*	Complement C3	2386	1763	623	187.0
7	P01859	*IGHG2*	Immunoglobulin heavy constant gamma 2	1612	1469	143	35.9
8	P01023	*α2MG*	Alpha-2-macroglobulin	2644	1660	984	163.2
9	P01860	*IGHG3*	Immunoglobulin heavy constant gamma 3	1531	1501	30	41.3
10	P0DOX5	*IGG1*	Immunoglobulin gamma-1 heavy	3021	2752	269	49.3

**Table 5:** Proteins significantly higher in db vitreous EVs.

#	Accession Number	Protein	Symbol	*p* Value
1	P43320	Beta-crystallin B2	*CRYBB2*	1.39 × 10^−6^
2	P02511	Alpha-crystallin B chain	*CRYAB*	2.33 × 10^−6^
3	P02489	Alpha-crystallin A chain	*CRYAA*	3.75 × 10^−5^
4	P53674	Beta-crystallin B1	*CRYBB1*	0.000575
5	P05813	Beta-crystallin A3	*CRYBA1*	0.001324
6	P22914	Gamma-crystallin S	*CRYGS*	0.008679
7	A0A075B6N3	T cell receptor beta variable 24-1	*TRBV24-1*	0.008733
8	Q8WXI7	Mucin-16	*MUC16*	0.012206
9	O95171	Sciellin	*SCEL*	0.024892
10	Q2TB90	Hexokinase	*HKDC1*	0.025755

**Table 6:** Proteins significantly lower in diabetic human vitreous samples.

#	Accession Number	Protein	Symbol	*p* Value
1	P04406	Glyceraldehyde-3-phosphate dehydrogenase	*GAPDH*	0.046
2	P12277	Creatine kinase B-type	*CKB*	0.040
3	P14618	Pyruvate kinase PKM	*PKM*	0.024
4	Q9UN88	Gamma-aminobutyric acid receptor subunit theta	*GABRQ*	0.012

**Table 7:** Association of db vitreous EV deregulated proteins with diseases.

#	Disease Name	*p*-Value
1	Cataract	5.805 × 10^−10^
2	Blindness	7.237 × 10^−8^
3	Presbyopia	0.0002650
4	Cystic fibrosis	0.005389
5	Dressler’s syndrome	0.006732

## Data Availability

All the data are included in the manuscript and the raw data are included in the Supplemental File.

## Supplementary Materials

The following supporting information can be downloaded at: https://www.mdpi.com/article/10.3390/biomedicines12092053/s1, Table S1: Multi-consensus proteomic analysis of vitreous EVs.
